# A RNA-seq approach to identify putative toxins from acrorhagi in aggressive and non-aggressive *Anthopleura elegantissima* polyps

**DOI:** 10.1186/s12864-015-1417-4

**Published:** 2015-03-21

**Authors:** Jason Macrander, Mercer R Brugler, Marymegan Daly

**Affiliations:** The Ohio State University, Evolution, Ecology, and Organismal Biology, 318 W. 12th Avenue, Columbus, OH 43210-1293 USA; Sackler Institute for Comparative Genomics, Division of Invertebrate Zoology, American Museum of Natural History, Central Park West at 79th Street, New York, NY 10024 USA; Biological Sciences Department, NYC College of Technology (CUNY), 300 Jay Street, Brooklyn, NY 11201 USA

**Keywords:** Phospholipase A2, Cytolysin, Sodium channel toxin, Potassium channel toxin, Acrorhagins, Venom, Cysteine, Neurotoxins, Intraspecific Competition

## Abstract

**Background:**

The use of venom in intraspecific aggression is uncommon and venom-transmitting structures specifically used for intraspecific competition are found in few lineages of venomous taxa. Next-generation transcriptome sequencing allows robust characterization of venom diversity and exploration of functionally unique tissues. Using a tissue-specific RNA-seq approach, we investigate the venom composition and gene ontology diversity of acrorhagi, specialized structures used in intraspecific competition, in aggressive and non-aggressive polyps of the aggregating sea anemone *Anthopleura elegantissima* (Cnidaria: Anthozoa: Hexacorallia: Actiniaria: Actiniidae).

**Results:**

Collectively, we generated approximately 450,000 transcripts from acrorhagi of aggressive and non-aggressive polyps. For both transcriptomes we identified 65 candidate sea anemone toxin genes, representing phospholipase A2s, cytolysins, neurotoxins, and acrorhagins. When compared to previously characterized sea anemone toxin assemblages, each transcriptome revealed greater within-species sequence divergence across all toxin types. The transcriptome of the aggressive polyp had a higher abundance of type II voltage gated potassium channel toxins/Kunitz-type protease inhibitors and type II acrorhagins. Using toxin-like proteins from other venomous taxa, we also identified 612 candidate toxin-like transcripts with signaling regions, potentially unidentified secretory toxin-like proteins. Among these, metallopeptidases and cysteine rich (CRISP) candidate transcripts were in high abundance. Furthermore, our gene ontology analyses identified a high prevalence of genes associated with “blood coagulation” and “positive regulation of apoptosis”, as well as “nucleoside: sodium symporter activity” and “ion channel binding”. The resulting assemblage of expressed genes may represent synergistic proteins associated with toxins or proteins related to the morphology and behavior exhibited by the aggressive polyp.

**Conclusion:**

We implement a multifaceted approach to investigate the assemblage of expressed genes specifically within acrorhagi, specialized structures used only for intraspecific competition. By combining differential expression, phylogenetic, and gene ontology analyses, we identify several candidate toxins and other potentially important proteins in acrorhagi of *A. elegantissima.* Although not all of the toxins identified are used in intraspecific competition, our analysis highlights some candidates that may play a vital role in intraspecific competition. Our findings provide a framework for further investigation into components of venom used exclusively for intraspecific competition in acrorhagi-bearing sea anemones and potentially other venomous animals.

**Electronic supplementary material:**

The online version of this article (doi:10.1186/s12864-015-1417-4) contains supplementary material, which is available to authorized users.

## Background

**Figure 1:**
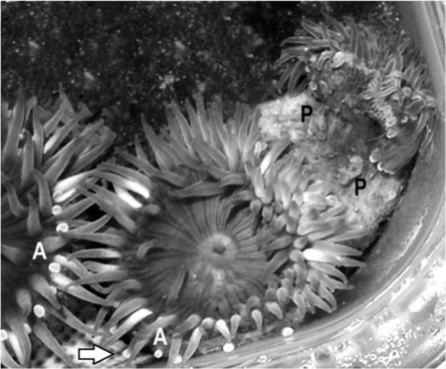
**Intraspecific aggression in** ***Anthopleura elegantissima*** **polyps.** Acrorhagi (A) are visible on the polyp in the center and the polyp to the left. A highly extended acrorhagus of the central polyp (arrow) is being applied to the column of the polyp on the left. Unlike the filiform tentacles, acrorhagi are opaque and rounded at the tip, even in extension. The polyp on the right has contracted in response to its encounter with the polyp in the center; its column is covered with mucus and several acrorhagial peels (P) from the central polyp.

Venomous animals use specialized structures to transmit a cocktail of noxious peptides into other organisms for defense or predation. Although the use of venom for intraspecific competition has been implied [1,2], specific examples of venom used on conspecifics via an intraspecific venom delivery system are rare [3]. Sea anemones are thus unique among venomous animals, as many species participate in intraspecific aggressive encounters using specialized structures to transmit venom [4-7]. Within the family Actiniidae, several species engage in aggressive intraspecific encounters using structures called acrorhagi [5,7]. Acrorhagi are inflatable structures at the tentacle-column margin that bear holotrichous nematocysts (reviewed in [8]). During an aggressive encounter, the acrorhagi inflate and adhere to the opponent (Figure 1), leaving an acrorhagial peel on the opponent [9]. The whitish peel, aggressor’s acrorhagi adhering to the victim (Figure 1), is surrounded by necrotic tissue [10].

In the sea anemone *Anthopleura elegantissima* (Actiniaria: Actiniidae), fierce competition for space in the coastal intertidal zone may have selected for strategies and behaviors that provide an advantage in intraspecific aggressive encounters [11-13]. These animals form dense clonal aggregations of asexually produced polyps that are physically distinct but closely spaced. Those polyps at the boundary of a clonal aggregation have a high number of acrorhagi proportionate to body size and often show signs of localized necrosis from acrorhagial peels of nearby non-clonemate anemones [13]. Acrorhagi-induced necrosis in *A. elegantissima* may be the result of an autoimmune process by which the allogeneic acrorhagial peel is isolated and expelled or may be caused by acrorhagi-specific toxins and necrosis-inducing compounds. The frequency of acrorhagial application is greater in intraspecific interactions than in interspecific interactions [5], highlighting their importance in intraspecific competition. The ectoderm of an acrorhagus generally does not adhere to the body of its bearer and the structure is not activated during prey capture, suggesting that the stimulus for the reaction and the discharge of nematocysts is “not-self” chemical signals. The mechanism behind the localized necrosis at the molecular level remains unknown; however, acrorhagi have been shown to transmit venom [14] and other bioactive components [15].

Toxins that have been well characterized within sea anemones fall into three major classes: phospholipase A2s (PLA2s), cytolysins, and neurotoxins. Within each class, several types (or groups) have been described based on sequence similarity and pharmacological target [16-19]. PLA2 genes belong to a large gene family whose members play varied roles in membrane remodeling, localized inflammation, and cell membrane, lipid, and amino acid metabolism [20-23]. The functional role of PLA2s has been studied in several cnidarians [17,24,25]; in some of these cases, PLA2 activity is associated with skin irritation in humans **(**eg. *Millepora* sp., see [25]). Group I and II PLA2s have been labeled as functionally toxic; along with an unknown venom component, they hydrolyze phospholipids and disrupt the cell membrane [26,27].

Although classified into four paralogous groups, all cytolysins form pores in the cellular membrane, creating an ionic imbalance that results in cytolysis [18,28-31]. Unlike other classes of toxins discussed here, cytolysins do not have disulfide bonds, relying instead on several amino acid residues for proper folding [18,32]. In term of function, cytolysins are ideal candidate agents for the localized necrosis observed in the victim of an intraspecific aggressive encounter, however, they cannot form pores in cnidarian cells because cnidarians lack the target lipid sphingomyelin in their cell membranes [18,33,34].

Neurotoxins, specifically voltage gated potassium channel **(**VGPC**)** and voltage gated sodium channel **(**VGSC**)** toxins, target residues on voltage gated ion channels, disrupting the normal flux of ions in to or out of the cell [35,36]. Diverse animal toxin genes target components within the VGPC and VGSC, including the elements that filter, activate, and close these channels [37-40]. VGPC are the most diverse type of ion channel [41], regulating a variety of cellular processes and functions by permitting the efflux of potassium ions across the membrane in response to cellular depolarization [16]. VGSC are transmembrane complexes consisting of four homologous domains, each of which comprises six subunits that span the cellular membrane [42]. The VGSC enables the initiation and propagation of action potentials through a rapid release of sodium ions across the cell membrane [43].

Unlike other venomous animals, cnidarians lack a centralized venom gland. This may permit the evolution of specialized venom cocktails in association with specific tissues or structures. *Adamsia carciniopados***(**Actiniaria: Hormathiidae**)** is the only sea anemone in which toxin activity was analyzed in multiple tissues; in this species, acontia showed higher phospholipase A2 activity than tentacles or whole body extracts [44]. Unfortunately, knowledge about the occurrence of functionally important venom in specific tissue types is rare: the majority of sea anemone toxins have been characterized from either whole animal or tentacle extracts (see Table six in reference [45]).

We characterize the diversity and abundance of toxins and potentially important peptides within the acrorhagi of *A. elegantissima*. Acrorhagi-specific toxins involved in intraspecific competition have been explored previously in *Actinia equina* (Actiniaria: Actiniidae) through a combined protein sequencing and RT-PCR approach [14], resulting in the identification of two candidate peptide toxins (acrorhagins). We sequenced RNA from acrorhagi of a single aggressive polyp and the (pooled) RNA from acrorhagi of several non-aggressive polyps. We screened each transcriptome for toxin genes using structural bioinformatics and phylogenetics. Gene networks of candidate toxin genes were used to investigate evolutionary patterns of gene diversity. We annotated candidate genes to highlight differences between the transcriptomes of acrorhagi from aggressive and non-aggressive polyps and provide insight into the putative function of acrorhagi.

## Results and discussion

### Next-Gen sequencing

**Figure 2:**
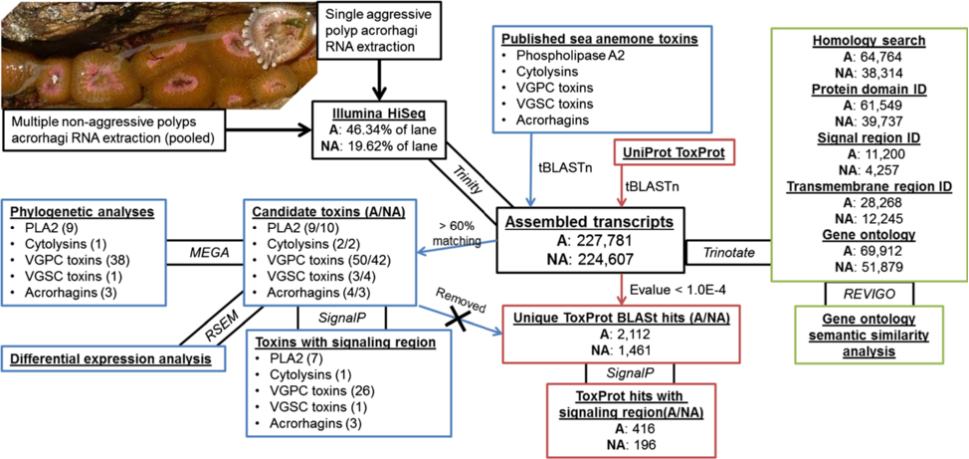
**Analytical pipeline for acrorhagi transcriptomes.** Colored boxes correspond to subsets of analyses with results reported in the text specific to sea anemone venom (blue), the UniProt animal toxin annotation, ToxProt (red), or transcriptome annotation, Trinotate (green). Text connecting these boxes indicate the analytical program used. Arrows with text indicates BLAST search strategies or thresholds used in initial screening.

Our approach provides a comprehensive view of acrorhagi-specific venom toxins within aggressive and non-aggressive polyps of *A. elegantissima*. Each transcriptome was subjected to a suite of analyses to investigate the diversity of toxin-like sequences and overall gene ontology within acrorhagi of aggressive and non-aggressive polyps (Figure 2). Contrary to previous toxin specific studies using mass spectrometry, an RNA-seq approach permits the rapid identification of multiple toxins and their relative expression levels. We retrieve entire toxin transcripts, including the signal and propeptide region, which are cleaved in post translation modification. Similar approaches have been used for studies of the venom gland or duct of non-cnidarian venomous taxa [40,46,47]. Because cnidarians do not have a specialized venom gland, the majority of genes in our transcriptomes are presumably not involved in envenomation; the “acrorhagi” transcriptomes include transcripts from holotrichous nematocysts and adjacent cells that perform other functions. Although gland cells found within tentacles excrete components of sea anemone venom [48], their role in acrorhagi venom excretion has not been explored.

We found four unexpected outcomes in our comparison of the transcriptomes of acrorhagi from aggressive and non-aggressive polyps. First, the size of the transcriptome did not influence our ability to retrieve candidate toxin genes. The aggressive polyp transcriptome was more than double the size of the non-aggressive polyp transcriptome in terms of raw sequence number (Table 1); nonetheless, we recover similar numbers of candidate toxin genes in each (Table 2). Second, there were no toxin genes expressed exclusively at high levels in either the aggressive or non-aggressive polyp transcriptome, which suggests that toxin components used in intraspecific aggression may always be expressed at some baseline level or that they cannot be identified based on homology to known toxin sequences. Third, acrorhagi contain a tremendous diversity of toxins. It is unlikely that every toxin gene is necessary for intraspecific aggression (discussed below); however, the array of toxin genes in acrorhagi of aggressive and non-aggressive polyps suggests that toxin genes may be expressed at some baseline level in most nematocysts or in glandular cells regardless of tissue, that there are alternative tissue specific forms for each class of toxin, or that acrorhagi retain some genes that reflect their “pre-acrorhagi” origin. Finally, despite having high sequence diversity and abundance among toxin genes, the frequency of multiple alleles or isoforms among our transcripts is relatively low compared to previous EST approaches in sea anemones [49-51]. It is likely that similar isoforms being expressed at low levels were incorporated into a single transcript [52].

**Table 1:** **Summary of sequencing, cleanup, and assembly**

**Sequencing**	**Aggressive**	**Non-aggressive**
**Total reads (% of Lane)**	**189,025,968 (46.34** **%)**	**83,914,158 (19.62** **%)**
**Trimmomatic cleanup**		
PE recovered (% of reads)	90,9451,67 (96.23%)	41,957,079 (96.33%)
Forward only (% of reads)	2,993,563 (3.17%)	1,257,747 (3.00%)
Reverse only (% of reads)	263,778 (0.27%)	192,526 (0.46%)
Dropped (% of reads)	310,476 (0.33%)	90,639 (0.22%)
**Trinity Assembly**		
Transcripts	227,781	224,607
Components/‘genes’	150,270	184,129
N50	1832	961

PE = Paired End. Modified sequences used in analyses can be found in Additional file 1.

**Table 2:** **Summary of candidate sea anemone toxin sequences**

	**A**	**NA**	**N**	**C**	**S**
**Phospholipase A2**	9	10	10	9	7
**Cytolysins**					
*Type II*	1	1	1	1	0
*Type IV*	1	1	1	-	1
**VGPC Toxins**					
*Type I*	9	10	13	11	8
*Type II*	19	12	14	13	4
*Type III*	19	17	17	14	12
*Type IV*	1	1	1	-	0
*Type V*	2	2	2	-	2
**VGSC Toxins**					
*Type I, II, & III*	3	4	3	3	1
**Acrorhagins**					
*Type I*	3	2	2	-	3
*Type II*	1	1	1	-	0
**Total**	**69**	**59**	**65**	**42**	**38**

A = aggressive polyp acrorhagi, NA = non-aggressive polyp acrorhagi, N = number of new toxin candidates (>90% similarity), C = number of gene clusters (>50 bootstrap support), S = genes with a signaling region; − = no phylogenetic reconstruction done.

### Candidate toxin genes

**Figure 3:**
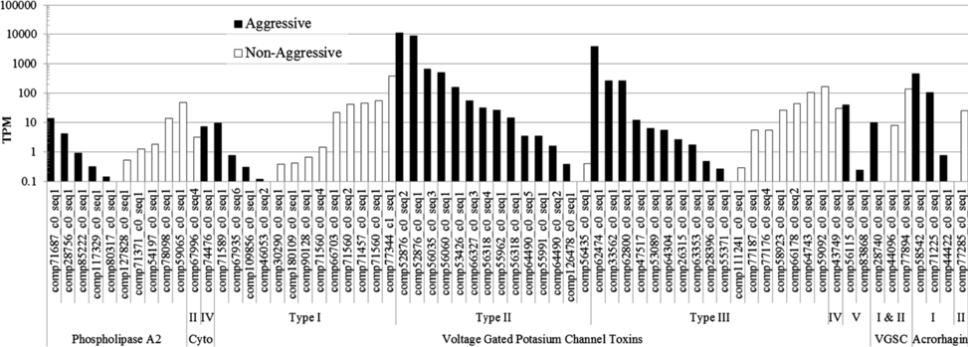
**Expression level differences among sea anemone toxin candidates in acrorhagi of aggressive and non-aggressive polyps.** Expression level expressed as ‘transcripts per million’ (TPM) for each transcriptome and compared using a logarithmic scale. For each gene, transcript names with the higher TPM values are shown.

By combining BLAST [53] searches with structural bioinformatics and toxin gene networks, we identified 65 candidate toxin genes that belong to five classes (Table 2; Additional file 1). We differentiate the relative levels of expression for each of these in each transcriptome, highlighting higher levels of expression in the acrorhagi of the aggressive polyp for the type II VGPC toxins/Kunitz-type protease inhibitors and type I acrorhagins (Figure 3). The newly-identified candidate toxin genes include 10 PLA2s, two cytolysins, 47 VGPCs, three VGSCs, and three acrorhagins. Of the 65 toxin genes identified, 38 included the start codon and signaling region (Table 2). Due to low levels of sequence variation, we could not differentiate among type I, II or III VGSC toxins based on sequence similarity alone. For many toxin types, our data contributed a large (>25%) proportion of sequences in each respective toxin gene network (PLA2 and types I – III VGPC toxins). This likely reflects our RNA-seq approach, rather than any intrinsic property of the focal tissue or taxon. Alignment lengths of the mature toxin residues varied considerably between the candidate toxins (41 – 467; Additional file 2: Table S1). In cases where taxonomic diversity was limited for toxin genes (type IV cytolysins, acrorhagins, types IV and V VGPC toxins), we did not conduct a gene network analysis. For the remaining toxin types (PLA2s, type II cytolysins, type I-III VGPC toxins, and VGSC toxins), we conducted gene network reconstructions with our new candidate toxin genes in combination with previously described toxins. Gene network reconstructions permitted the grouping of isoforms into different genes based on high levels of sequence similarity. Even though the isoforms clustered, their high sequence divergence suggests several new toxin gene candidates for PLA2s (9), type I (11), type II (13), and type III (14) VGPC toxins (Table 2).

### Phospholipases A2

**Figure 4:**
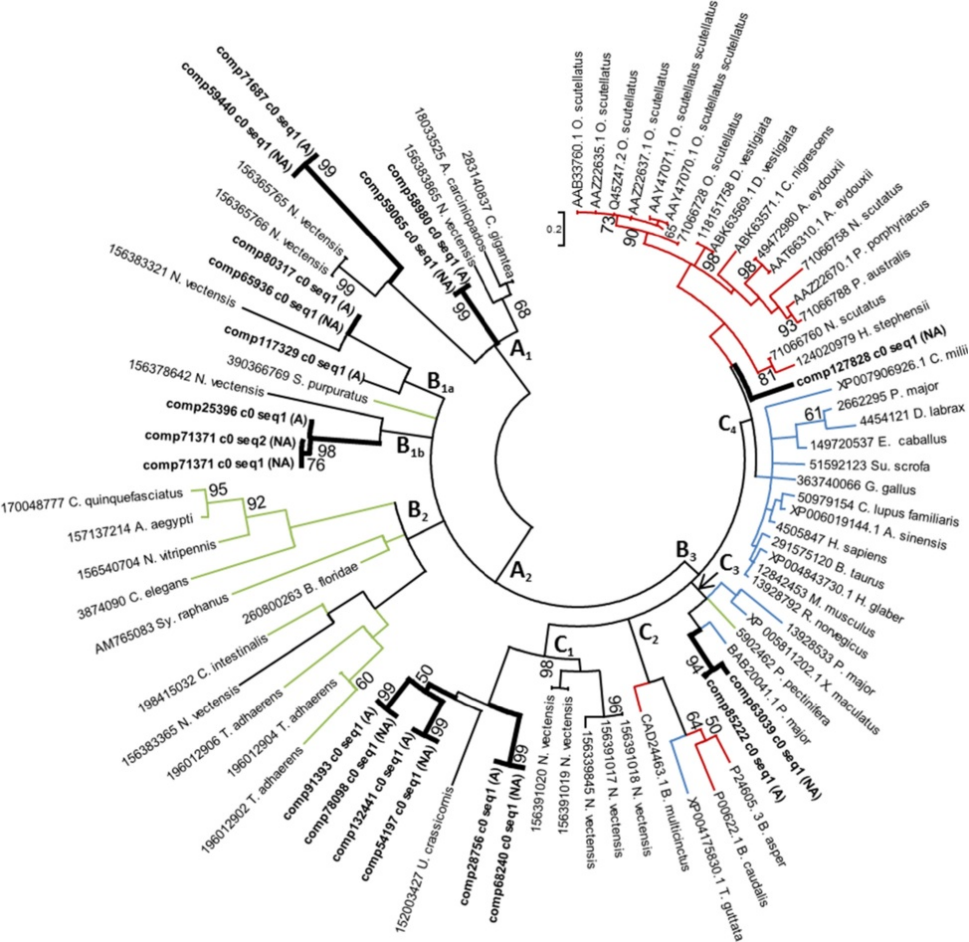
**Maximum likelihood gene network of PLA2 genes.** Color of the branches indicate function and origin: non-venomous PLA2 genes from non-cnidarian invertebrates (green, B_1_ [*S. purpuratus*: sea urchin] and B_2_ [*A. aegypti & C. quinquefasciatus*: mosquito; *B. floridae*: lancelet; *C. elegans*: nematode; *C. intestinalis*: tunicate; *N. vitripennis*: parasitoid wasp; *Sy. raphans*: sponge; *T. adhaerens*: placozoan]); PLA2 toxins found in vertebrates (red, C_2_ [*B. asper*, *B. caudalis, & B. multicinctus*: snakes] and C_4_ [*A. eydouxii*, *C. nigrescens*, *D. vestigiata*, *H. stephensii N. scutatus*, *O. scutellatus*, *P.* porphyriacus, & *P. australis*: snakes]); non-venomous PLA2 genes found in vertebrates (blue, C_1_ [*T. guttata*: zebra finch] C_3_ [*P. major & X. maculatus*: fish] and C_4_ [*A. sinensis*: alligator; *B. taurus*: cattle; *C. millii*: elephant fish; *C. lupus familiaris*: dog; *D. labrax & P. major*: fish; *E. caballus*: horse; *G. gallus*: chicken; *H. glaber, M. musculus, & R. norvegicus*: rodents; *H. sapien:* human; *Su. scrofa*: pig]) and an invertebrate (green C_3_ [*P. pectinifera*: sea star]; and PLA2 genes from sea anemones which may or may not be venomous (black, A_1_ and A_2_). Newly-identified candidate toxin genes are in bold with thick branches and the source is indicated (acrorhagi from A: aggressive or NA: non-aggressive polyps). Labels at the terminal tips indicate GenBank accession number and species identity. For full species names refer to S. Table 2. Bootstrap support values greater than 50 are shown.

Toxin gene networks were reconstructed for the mature PLA2 gene sequences from acrohagi, previously characterized sea anemone PLA2s, and PLA2s from venomous and non-venomous vertebrate and non-vertebrate taxa (Figure 4). The resulting gene network suggests that *A. elegantissima* has at least six kinds of PLA2 in the acrorhagi, with some having multiple genes clustered together (Figure 4). At least three of these are part of a cluster that includes only genes from sea anemones (A_1_, B_1a_, B_1b_: Figure 4). The remaining genes are part of a complex network of genes from vertebrates and non-vertebrate species (including other sea anemones) that are associated with venoms and not known to have toxic properties (A_2_: Figure 4).

Within this complex network, PLA2 genes from the model organism *Nematostella vectensis* are associated with genes from the urochordate *C. intestinalis* and the placozoan *T. adhaerens* (see B_2_: Figure 4). No sequences from our transcriptomes belong to this clade. The remaining sea anemone PLA2 genes nest within a cluster of genes from vertebrates (B_3_: Figure 4). This gene cluster forms a large polytomy with a sea anemone exclusive cluster (C_1_: Figure 4); a non-venomous vertebrate gene cluster that also contains a candidate sea anemone PLA2 gene (C_3_: Figure 4); a mixed venomous and nonvenomous PLA2 gene cluster of vertebrates (C_2_: Figure 4); and a cluster that contains genes with both venomous and non-venomous function with a single incomplete *A. elegantissima* candidate PLA2 toxin gene (C_4_: Figure 4). The polytomies and relatively low bootstrap support for groups within this network likely reflect incomplete taxon sampling [54]. Nonetheless, the placement of the sea anemone PLA2 genes throughout the network is worth noting: sea anemone PLA2 genes (or clusters of genes) lie outside the split between vertebrates and non-vertebrates, as would be predicted based on organismal phylogeny and also lie within a cluster of genes from vertebrates, suggesting an ancestral gene duplication event with subsequent gene loss. The network of PLA2s is complex in terms of the phylogenetic affinities of various members of the gene family and the nature of these in terms of their known use as venoms. Denser sampling of taxa and of copies within these taxa is necessary to understand the evolution and diversification of this gene family.

### Cytolysins

**Figure 5:**
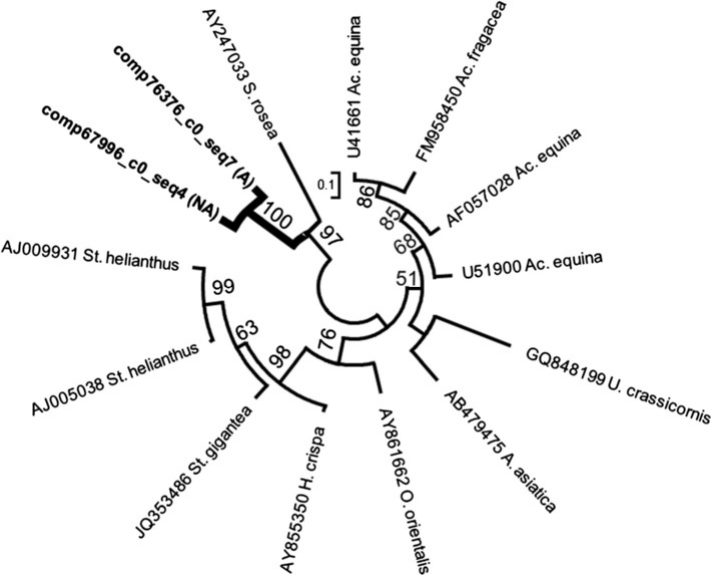
**Maximum likelihood gene network of the type II cytolysin genes.** Newly-identified candidate toxins are in bold with thick branches. The transcriptome source is indicated (acrorhagi from A: aggressive or NA: non-aggressive polyps). Labels at the terminal tips indicate GenBank accession number and species identity. For full species names refer to S. Table 2. Bootstrap support values greater than 50 are shown.

We found one of each type II and type IV candidate cytolysin toxins in the transcriptomes of acrorhagi from aggressive and non-aggressive polyps. Due to the small number of type IV cytolysins previously identified, we did not construct a gene network for this toxin. In the gene network for type II cytolysin toxins, our new candidate gene from the acrorhagi of *A. elegantissima* groups as the sister to a cytolysin from *Sagartia rosea* (Figure 5), a finding that was supported whether we considered protein or nucleotide sequences. The relationship inferred from the cytolysin genes is at odds with the phylogeny of sea anemones [55]: the acrorhagi-bearing species *Ac. equina* (and *Anthopleura asiatica*) are more closely related to *A. elegantissima* than is the metrideoidean *S. rosea*. The divergence observed in our candidate acrorhagi cytolysins is worth noting, as previously described cytolysins of *Ac. equina* were extracted from whole specimens, not acrorhagi [56,57]. Strong selective pressures or potentially loss of function could explain the observed divergence between the *A. elegantissima* cytolysin and *Ac. equina* cytolysin isolated from whole specimens. The high level of sequence variation may be indicative of a highly divergent or paralogous type II cytolysin in the acrorhagi of *A. elegantissima* that has undergone neofunctionalization or alternatively is losing its cytolytic potency. Whether this toxin still targets sphingomyelin, another lipid, or has completely lost its cytolytic function cannot be determined based on sequence data alone.

### Voltage gated potassium channel toxins

In sea anemones, toxins that target components of the voltage gated potassium channel (VGPC) are interpreted to be diverse in origin and function, compared to those targeting the voltage gated sodium channel (VGSC) [45]. Sea anemone VGPC toxins have been classified into five types (I-V) based on amino acid composition, folding pattern, and target site [19,45,50,58]. We find candidate genes belonging to each type in the transcriptomes of acrorhagi from aggressive and non-aggressive polyps.

**Figure 6:**
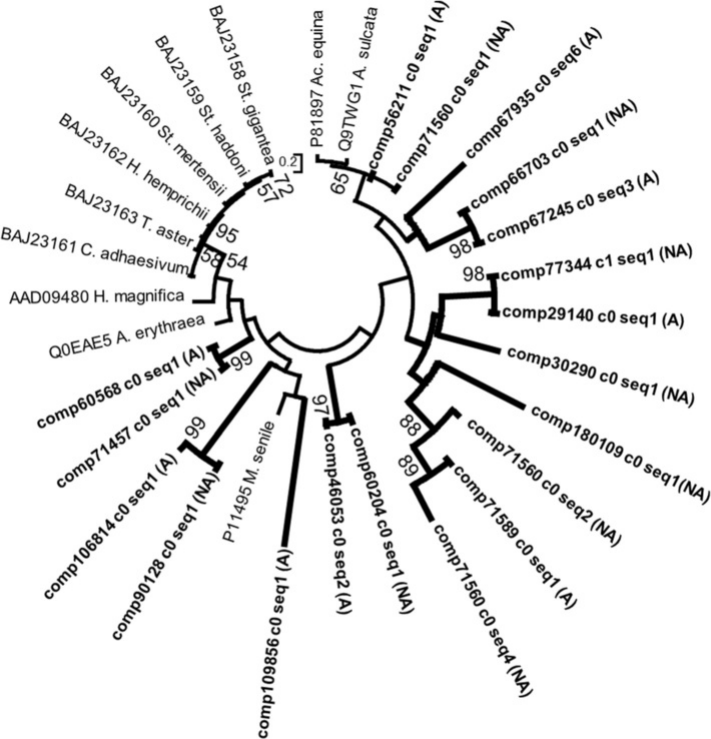
**Maximum likelihood gene network of the type I VGPC toxin genes.** Newly-identified candidate toxins are in bold and the source is indicated (acrorhagi from A: aggressive or NA: non-aggressive polyps). Labels at the terminal tips indicate GenBank accession number and species identity. For full species name refer to S. Table 2. Bootstrap support values greater than 50 are shown.

We identify 13 candidate genes that correspond to type 1 VGPC toxin genes based on shared sequence identity, conserved cysteine residues, and toxin gene network reconstruction (Table 2). The network for type I VGPC genes had relatively low support values throughout, with 11 candidate gene clusters having >50% bootstrap support values (Figure 6). Although the cysteine residues are maintained across our toxin candidates, the rate of amino acid substitution throughout the candidate toxin genes exceeds what has been reported previously (Additional file 2: Figure S1). In addition to these 13 candidate VGPC genes, we found several transcripts containing multiple (rather than single) type I VGPC toxin-like domains. These transcripts may also be type I VGPC toxins, as other toxins have been shown to go through post translational modifications [59].

**Figure 7:**
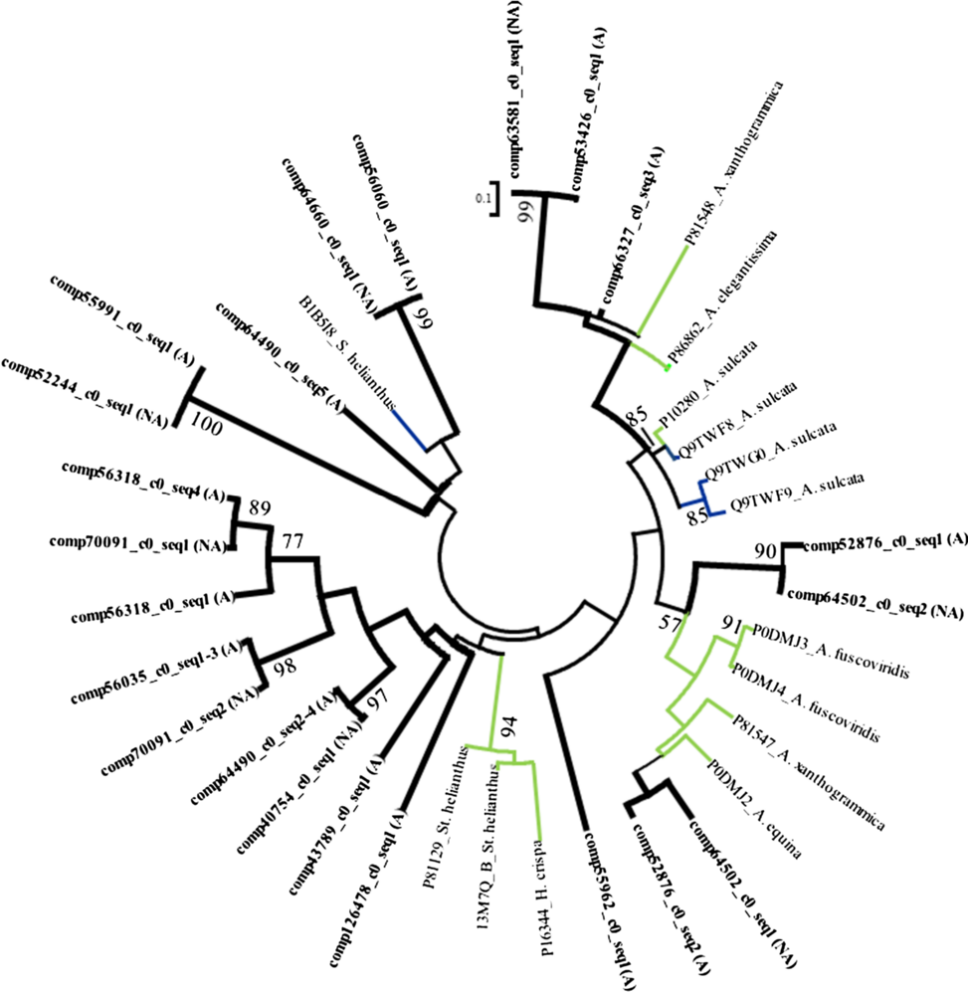
**Maximum likelihood gene network of type II VGPC toxin/Kunitz protease inhibitor genes.** Previously characterized protease inhibitors (green) and type II VGPC toxins (blue) are highlighted. Newly-identified candidate toxins are in bold. The transcriptome source is indicated (acrorhagi from A: aggressive or NA: non-aggressive polyps). Labels at the terminal tips indicate GenBank accession number and species identity. For full species names refer to S. Table 2. Bootstrap support values greater than 50 are shown.

Type II VGPC toxins are dual functioning, acting as VGPC blockers and Kunitz-type protease inhibitors (KPI). The role of KPI in sea anemone venom is not completely understood. They are inferred to inhibit endogenous proteases in their targets or to protect the toxins against proteases following target injection [36]. KPI may have adopted VGPC blocking activity following slight changes in amino acid residues [60]. KPI have been recruited into venom in diverse lineages through convergent evolution [1]. From the sequence alignments, we were unable to distinguish between type II VGPC toxins and KPI and included both in our type II VGPC toxin network reconstruction. In this network, many KPI genes form well-supported groups (Figure 7); still other KPI genes outside of these clusters were found alongside known type II VGPC toxins. KPI and type II VGPC toxins show close affinity and this evidence of acquisition of VGPC blocking: sequences of known venom function from *Anemonia sulcata* are sister to genes that have KPI-type function (Figure 7). The lack of separation between type II VGPC toxins and KPI genes could reflect strong selection pressures needed to maintain KPI function, rather than convergence towards VGPC blocking activity. Regardless of cause, this close affinity between KPI and type II VGPC genes makes predicting the function of our candidate sequences difficult: based on the tree, it is impossible to determine whether the diversification represents a series of candidate type II toxins or a diversification of KPI genes or a combination of the two (Figure 7).

The majority of the type II VGPC/KPI candidates were expressed at higher levels in the acrorhagi of aggressive polyps (Figure 3). The two type II VGPC/KPI candidate toxins with the highest TPM values (comp64502_c0_seq1 & comp52876_c0_seq2) were part of a clade that contained genes that lack KPI function (Figure 7), which likely indicate that they are functionally KPI genes rather than both KPI and VGPC toxins. Although type II VGPC toxins are also functionally KPI genes [61], gene expression levels do not necessarily predict functional importance [62]. The high expression candidate KPI genes identified here may behave synergistically with functionally important toxins following target injection (i.e. the type II cytolysins and acrorhagins discussed below), rather than targeting the VGPC. The role of type II VGPC toxins as synergistic KPI proteins needs to be explored with regards to intraspecific envenomation, as well as their use in predation or defense.

**Figure 8:**
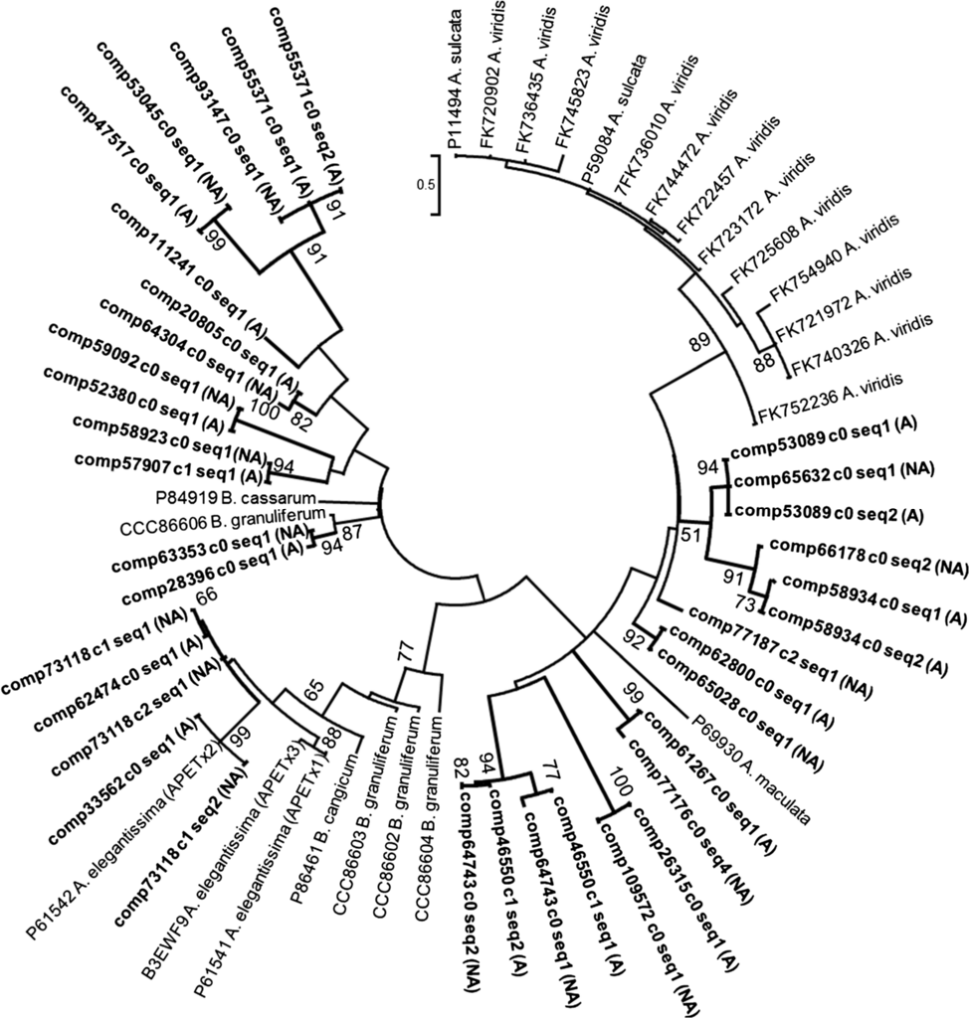
**Maximum likelihood gene network of the type III VGPC toxin genes.** Newly-identified candidate toxins are in bold and the source is indicated (acrorhagi from A: aggressive or NA: non-aggressive polyps). Labels at the terminal tips indicate GenBank accession number and species identity. For full species names refer to S. Table 2. Bootstrap support values greater than 50 are shown.

Type III VGPC toxins are similar to VGSC toxins based on the placement of structurally important cysteine residues, but they show no effect on VGSC [36]. Type III VGPC toxins in sea anemones vary considerably in their function and have been described as having multiple target sites [39,63,64]; this could provide plasticity among targets [62] and allow these toxins to modify more than just VGPC [16]. Our toxin gene network for type III VGPC toxins includes the acid sensing channel toxin (APETx2), as there were high levels of sequence similarity shared between this and other type III VGPC toxins. The toxin gene network has several new candidate type III VGPC toxins clustered with toxin genes previously characterized in *A. elegantissima* and in *Bunodosoma granuliferum, B. cangicum*, and *B. cassarum* (Figure 8). The type III VGPC toxin genes from *Anemonia viridis* and *An. sulcata* form a cluster apart from those from *Bunodosoma* and *Anthopleura* (Figure 8). The clustering of type III VGPC genes from the acrorhagi of *A. elegantissima* suggests that there are at least 13 forms of this toxin, including APETx2 genes (Figure 8).

Type IV VGPC toxins are relatively short, containing only two disulfide bonds and have been identified in only two species of sea anemones [65,66]. We recovered a single type IV toxin candidate gene that was represented in both transcriptomes (Accession: GBXJ01030381); however, the transcript was incomplete and did not have a signaling region (Table 2).

The type V VGPC toxins appear to be relatively conserved and have been found in distantly related species of Actiniaria [58]. We identified two candidate genes for type V VGPC toxins (Accession: GBXJ011150398 & GBXJ01058837); each of these has a unique signaling region (Table 2). The candidate toxin genes from each transcriptome had different amino acid sequences, which could be due to multiple alleles incorporated into a single transcript [52].

### Voltage gated sodium channel toxins

**Figure 9:**
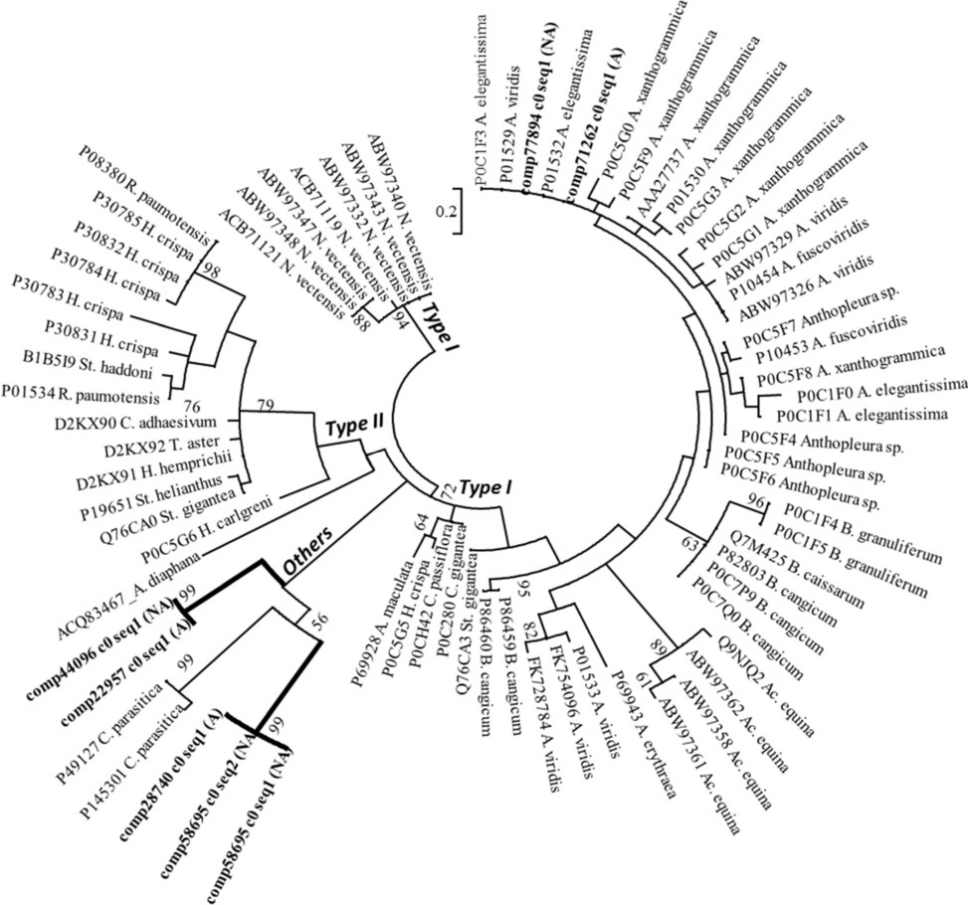
**Maximum likelihood gene network of the VGSC toxin genes.** The different toxin types (Type I, Type II, and “others”) are noted. Newly-identified candidate toxins are in bold and the source is indicated (acrorhagi from A: aggressive or NA: non-aggressive polyps). Labels at the terminal tips indicate GenBank accession number and species identity. For full species name refer to S. Table 2. Bootstrap support values greater than 50 are shown.

Like the VGPC, the VGSC is inferred to have been co-opted as a target in multiple venomous lineages [1,67]. VGSC toxins have been categorized into three types (type I, type II, and “others”), all of which have a similar arrangement of disulfide bonds [19,50]. VGSC toxins target multiple receptor sites in the sodium channel [35,45]. Because VGSC toxins have been a focal toxin in the study of sea anemones, our gene networks include toxins from several species, including a VGSC toxin previously described in *A. elegantissima* [68]. Our gene network reconstruction of the VGSC toxins found four distinct groups that correspond to those types previously identified as type I, type II, *N. vectensis* (type I), and “others” (Figure 9). In addition to the type I VGSC previously described from *A. elegantissima* [68,69], we find two unique VGSC candidate toxin genes. The type I genes we identified cluster with sequences previously identified from *A. elegantissima* (Figure 9), as well as other sequences from other species of *Anthopleura* and its allies (members of family Actiniidae). The unique VGSC candidates grouped outside the type I and type II VGSC genes, clustering instead with the “other" VGSC toxin genes from *Calliactis parasitica* (Figure 9). This is the first report of candidate VGSC toxin genes for endomyarian sea anemones clustering outside the type I and type II groupings [45].

### Acrorhagins

Acrorhagins were first described from acrohagi of *Ac. equina* and were originally thought to contribute to the phenotypic response observed in targets of intraspecific aggression [14]. Acrorhagins have also been isolated from acrorhagi of *Anthopleura xanthogrammica* and *A. fuscovirdis* [70]. Unlike those of *Ac. equina*, the acrorhagins of *A. xanthogrammica* and *A. fuscovirdis* exhibit no lethality to crabs [70]. The candidate acrorhagin I sequences from *A. elegantissima* are slightly longer (by 15 – 20 AA) than those from *Ac. equina* [14] but they have a similar signal peptide (Additional file 2: Figure S2). The candidate acrorhagin II sequences from *A. elegantissima* lacked any signaling region (Additional file 2: Figure S2). In contrast to the assumption that acrorhagins are unique to acrorhagi, we identify via reciprocal BLAST searches a candidate acrorhagin I toxin gene from an EST library from the sea anemone *Metridium senile* (Additional file 2: Figure S2). Although *M. senile* is distantly related and lacks acrorhagi, it does engage in aggressive intraspecific encounters with specialized structures containing holotrichous nematocysts [7].

### Other candidate toxin sequences

**Figure 10:**
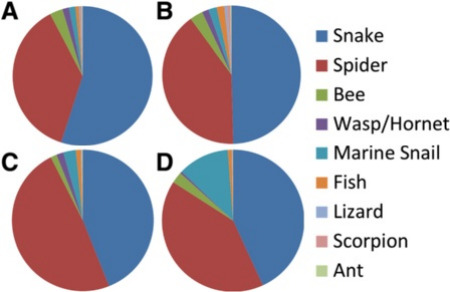
**ToxProt results by venomous organisms.** Proportion of different venomous groups identified when screening the ToxProt BLASTp hits for the aggressive (A + C) and non-aggressive polyp transcriptome (B + D) when considering the number of transcripts (A + B) or expression level as ‘transcripts per million’ (TPM) (C + D).

Through convergent evolution, selective processes have shaped the venom repertoire in many distantly related venomous taxa, often converting non-venomous proteins into venomous counterparts [reviewed in 1]. As a result, many distantly related taxa have toxins with similar functional residues, and these can be used to identify toxin gene candidates from transcriptome data [71]. We used BLAST to compare the transcriptomes of acrorhagi from aggressive and non-aggressive polyps to 5,938 annotated toxin protein sequences from the UniProt ToxProt dataset. We found 2,112 (aggressive polyp) and 1,461 (non-aggressive polyp) unique transcripts unique to the ToxProt sequences and would not have been identified by the searches that focused on sea anemone toxins alone. Of these, there were 416 (aggressive polyp) and 196 (non-aggressive polyp) with identified signaling regions (Additional file 3). The majority of the UniProt candidate toxin genes are from snakes and spiders (Figure 10).

Overall, the aggressive and non-aggressive polyp transcriptomes are similar in the genes and gene families recovered by BLAST searches against the UniProt ToxProt dataset (Figure 10A, B; Table 3), although the taxonomic association of the toxin genes shifted when ranked by level of expression (Figure 10C, D). Acrorhagi of an aggressive polyp have proportionally more expressed transcripts with significant hits from spider toxin genes (Figure 10C), specifically due to the TPM of the toxin Neprilysin-1, a metalloprotease. In contrast, those from the acrorhagi of non-aggressive polyps appear to have more transcripts associated with a snail toxin (Figure 10D), specifically Augerpeptide hhe53. Among the most highly expressed transcripts recovered in our BLAST analysis are the metalloprotease, CRISP, and peptidase proteins, with the majority of the highly expressed toxin-like gene types found in both the aggressive and non-aggressive polyp acrorhagi transcriptome BLAST results (Table 3). Although most transcripts identified here are likely non-venomous proteins of large gene families, the presence of a signaling region and high sequence similarity to other toxins indicates that there may be tremendous uncharacterized toxin diversity found in sea anemones.

**Table 3:** **Most highly expressed transcripts with significant ToxProt data BLAST hits**

**Aggressive polyp transcriptome**					
**transcript_id**	**TPM**	**ToxPROT**	**Group**	**Gene name (gene family)**	**E-value**
comp71163_c0_seq1	2734	G3LU44	Spider	Translationally-controlled tumor protein homolog (TCPT)	3.00E-08
comp29480_c0_seq1	1521	W4VS99	Spider	Neprilysin-1 (Metalloprotease)*	6.00E-64
comp68333_c3_seq1	1129	Q8AY75	Snake	Cysteine-rich venom protein pseudechetoxin (CRISP)	2.00E-34
comp71203_c0_seq1	903	P24541	Snake	Kunitz-type serine protease inhibitor (Kunitz-type)	3.00E-13
comp61501_c0_seq2	251	P35786	Wasp	Venom allergen 5 (CRISP)	1.00E-06
**comp61420_c0_seq1**	**227**	**J3S9D9**	**Snake**	**Ophiophagus venom factor (** ***NONE*** )*	**4.00E-40**
**comp67467_c0_seq2**	**175**	**P33589**	**Snake**	**Thrombin-like enzyme gyroxin analog (Peptidase S1)**	**2.00E-12**
**comp62144_c0_seq1**	**169**	**P0DM62**	**Spider**	**Astacin-like metalloprotease toxin 5 (Metalloprotease)**	**3.00E-28**
**comp67061_c0_seq19**	**164**	**P0DM61**	**Spider**	**Astacin-like metalloprotease toxin 4 (Metalloprotease)**	**3.00E-12**
**comp62592_c0_seq1**	**163**	**Q4PRD2**	**Snake**	Snaclec coagulation factor X-activating enzyme light chain 2 (Snaclec)*	**0.0001**
**Non aggressive polyp transcriptome**					
**transcript_id**	**TPM**	**ToxPROT**	**Group**	**Gene name (gene family)**	**E-value**
comp54814_c0_seq1	3051	G3LU44	Spider	Translationally-controlled tumor protein homolog (TCPT)	2.00E-03
comp71606_c0_seq1	1961	Q8AY75	Snake	Cysteine-rich venom protein pseudechetoxin (CRISP)	8.00E-35
comp43780_c0_seq1	1413	P0CI21	Snail	Augerpeptide hhe53 (*NONE*)*	0.00003
comp73391_c0_seq1	329	P24541	Snake	Kunitz-type serine protease inhibitor (venom Kunitz-type)	1.00E-13
**comp73577_c0_seq1**	**315**	**P0DM62**	**Spider**	**Astacin-like metalloprotease toxin 5 (Metalloprotease)**	**2.00E-28**
comp43784_c1_seq1	217	Q2XXR2	Lizard	Cysteine-rich venom protein VAR4 (Metalloprotease)*	0.00005
**comp43791_c0_seq1**	**202**	**P0C8M2**	**Snake**	Disintegrin ocellatin (Metalloprotease)*	**0.0001**
comp77542_c0_seq1	177	P43685	Wasp	Venom allergen 5 (CRISP)	2.00E-14
**comp73785_c0_seq2**	**170**	**P33589**	**Snake**	**Thrombin-like enzyme gyroxin analog (Peptidase S1)**	**3.00E-13**
comp66731_c0_seq1	136	Q8AY75	Snake	Cysteine-rich venom protein pseudechetoxin (CRISP)	1.00E-14

Transcripts are expressed as ‘transcripts per million’ (TPM); bold BLAST hits have an identified signaling region.

### Transcriptome characteristics and gene ontology

CEGMA [72] was used to assess the completeness of the acrorhagi from aggressive and non-aggressive polyps transcriptome [73,74]. Of the 248 core eukaryotic proteins, 245 (99%) in the aggressive polyp and 219 (88%) of the non-aggressive polyp acrorhagi transcriptomes were identified and considered complete (>70% alignment length with core proteins). There was an average of ~3.5 (aggressive polyp) and ~2.5 (non-aggressive polyp) orthologs per core protein. Despite having a large difference in the number of raw sequences for each transcriptome, both were deemed relatively complete by CEGMA.

The homology search identified 64,764 (aggressive polyp) and 38,314 (non-aggressive polyp) transcripts (Table 4, Additional file 4). Each of the BLASTx searches identified a greater proportion of transcripts when compared to the BLASTp searches for the transcriptome of acrorhagi from an aggressive (81,171 vs. 67, 648) and non-aggressive polyp (55,555 vs. 39,539) (Table 4, Additional file 4). The lower number of BLASTp matches for each transcriptome could be due to the incorrect ORF being translated in the Trinotate pipeline or reflect alternative search strategies between the two methods. The protein domain identification steps matched to the largest portion of the transcriptome, with the program HMMER using the Pfam database [75] identifying 53.37% (aggressive polyp) and 28.89% (non-aggressive polyp) of the transcripts as containing protein domains (Table 4, Additional file 4), with the smallest portions of the transcriptomes having signaling regions or transmembrane helices (Table 4, Additional file 4). Overall, Trinotate characterized only a small portion of the entire transcriptome (Table 4, Additional file 4), likely due to *A. elegantissima* being distantly related to the taxa populating the comparative databases.

**Table 4:** **Summary of Trinotate results**

	**Homology search**		**Protein domain ID**				**Gene ontology**		
	*BLASTx*	*BLASTp*	*HMMER*	*Pfam*	*SignalP*	*tmHMM*	*BP*	*MF*	*CC*
Aggressive	81,171	67,648	134,103	72,554	11,200	28,268	51,143	52,699	55,211
*% of Assembly*	*32.31*%	*26.92*%	*53.37*%	*28.88*%	*4.46*%	*11.25*%			
**Both**	**64,764**		**61,549**				**69,912**		
Non-aggressive	55,555	39,539	66,294	39,737	4,257	12,245	42,941	43,177	43,387
*% of assembly*	*24.21*%	*17.23*%	*28.89*%	*17.32*%	*1.86*%	*5.34*%			
**Both**	**38,314**		**39,737**				**51,879**		

Values represent number of transcripts and percent assembly during analysis. BP = Biological Process; MF = Molecular Function; CC = Cellular Components.

**Figure 11:**
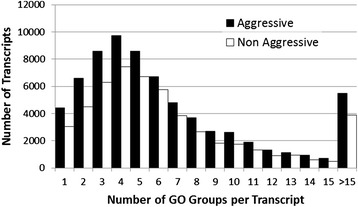
**GO terms per transcript.** Number of GO terms per transcript recovered through the Trinity pipeline.

The gene ontology annotation assigned 69,912 (aggressive polyp) and 51,879 (non-aggressive polyp) transcripts to at least one gene ontology group. There was a large discrepancy in the number of associated GO terms between the two transcriptomes: 469,339 (aggressive) and 359,316 (non-aggressive). This was due to the majority of sequences belonging to more than one gene ontology group (Figure 11). Although the CEGMA analysis revealed that the non-aggressive polyp acrorhagi transcriptome was less complete (99% vs 88%), the difference observed in these transcriptomes may be attributed to acrorhagial tissues being more transcriptionally active during an aggressive encounter. During the aggressive encounter, the acrorhagi of an aggressive polyp inflate, move, and respond to the target polyp, which likely involves a complex array of cellular signaling and metabolic processes not engaged in a non-aggressive polyp. Regardless, the transcripts of each transcriptome can be attributed to approximately 7900 different GO terms.

**Figure 12:**
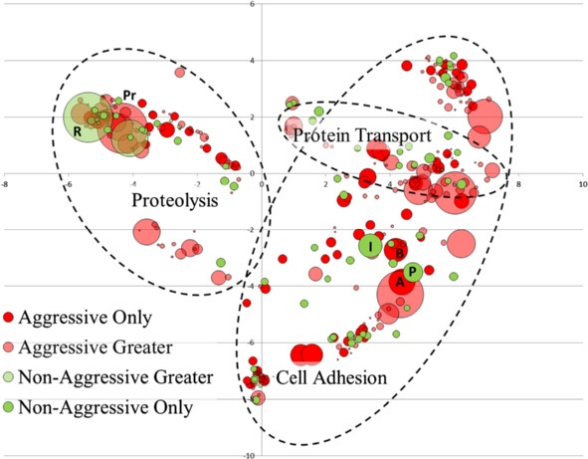
**Biological Process domain GO groupings for acrorhagi from aggressive and non-aggressive polyps.** Scatterplot clustering of transcripts representing Biological Process domain groupings in REVIGO. The x and y axis represent semantic coordinates in REVIGO; functionally similar gene ontology groups are closer together. Each colored circle represents a GO term, with its size proportionate to its assigned REVIGO value. Specific GO terms referenced in the text are noted: Pr: “proteolysis” (GO:0006508); R: “RNA-dependent DNA replication” (GO:0006278); B: “blood coagulation” (GO:0007596); P: “positive regulation of apoptosis” (GO:0043065); I: “induction of apoptosis” (GO:0006917); A: “platelet activation” (GO:0030168).

Overall, the patterns of association for transcripts were similar in the transcriptomes of acrorhagi from aggressive and non-aggressive polyps: most transcripts are associated with cellular components, followed by molecular function and biological process domains (Table 4). The REVIGO clustering algorithm identified semantic similarities among transcripts between groups by compressing the >7900 GO terms into representative lists of fewer than 350 for both the biological process and molecular function domains. All but 12 of the GO terms for the biological process domain grouped with cell adhesion, proteolysis, and protein transport (Figure 12), with the largest REVIGO value differences observed in the “proteolysis” (GO:0006508) GO group for the aggressive polyp transcriptome, and “RNA-dependent DNA replication” (GO:0006278) for the non-aggressive polyp transcriptome (Figure 12). Some GO groups with high REVIGO values were exclusively associated with the aggressive polyp transcriptome (“blood coagulation” GO:0007596 and “positive regulation of apoptosis” GO:0043065) and non-aggressive polyp transcriptome (“induction of apoptosis” GO:0006917 and “platelet activation” GO:0030168). These GO groups may contain toxin-like transcripts similar to synergistic components of snake venom which induce blood coagulation and affect platelet function [76].

**Figure 13:**
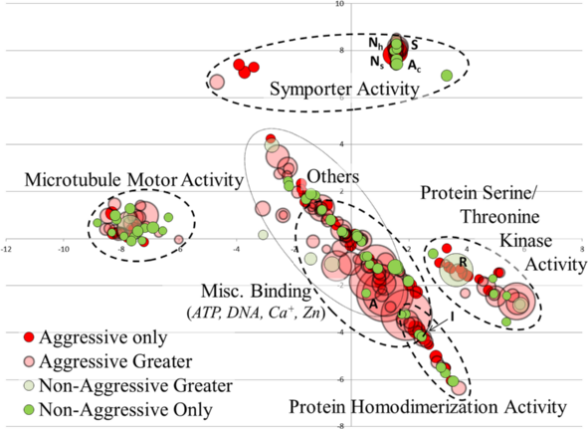
**Molecular Function domain GO groupings for acrorhagi from aggressive and non-aggressive polyps.** Scatterplot clustering of transcripts representing Molecular Function domain groupings in REVIGO. The x and y axis represent semantic coordinates in REVIGO; functionally similar gene ontology groups are closer together. Each colored circle represents a GO term, with its size proportional to the assigned REVIGO value. Specific GO terms referenced in the text are noted: A:ATP binding (GO:0005524); R: RNA-directed DNA polymerase activity (GO:0003964); N_s_: nucleoside:sodium symporter activity (GO:0005415); N_h_: nucleoside:hydrogen symporter activity (GO:0015506); I: ion channel binding; (GO:0044325), S: sodium channel activity; GO:0005272, A_c_: acetylcholine-activated cation-selective channel activity; GO:0004889.

Transcripts associated with GO groups in the molecular function domain were grouped into microtubule motor activity, symporter activity, miscellaneous binding, protein serine/threonine kinase activity, and protein homodimerization activity; the remaining 29 GO groups were highlighted as “others” (Figure 13). The largest differences in REVIGO values were observed in the “ATP binding” (GO:0005524) and “RNA-directed DNA polymerase activity” (GO:0003964) for the transcriptome of acrorhagi from aggressive and non-aggressive polyps, respectively (Figure 13). REVIGO groups with the highest values found exclusively in the aggressive polyp transcriptome were associated with the transfer of solutes across a cell membrane (“nucleoside:sodium symporter activity” GO:0005415 and “nucleoside: hydrogen symporter activity” GO:0015506) as well as ion channel function (“ion channel binding” GO:0044325, “sodium channel activity” GO:0005272, and “acetylcholine-activated cation-selective channel activity” GO:0004889) (Figure 13). These GO groups may be involved in disrupting homeostasis in target cells, thus behaving similarly to cytolysins or neurotoxins or gene products acting on the ion channels necessary for acrorhagi motility and movement.

## Conclusion

Venoms are rarely used exclusively in intraspecific competition, being more commonly employed in defense or predation against other species. We used transcriptomes of the acrorhagi from aggressive and non-aggressive polyps of the sea anemone *A. elegantissima* to investigate the venom proteins and peptides in a tissue specifically used in intraspecific competition. We found a diversity of genes associated with types I – III VGPC/KPI toxins and PLA2s; cytolysins and VGSC toxins were comparatively less diverse. The high number of candidate toxin genes we found is likely not specific to *A. elegantissima* or to acrorhagi, but reflects our next-generation sequencing approach and relatively sparse prior knowledge of genetic diversity of toxins in sea anemones. We found high sequence divergence among these toxin genes and hypothesize that some toxin alleles with low divergence were incorporated into a single transcript. Our study of cytolysins, type II VGPC/KPI toxins, VGSC toxins, and type I acrorhagins all produced unexpected results in terms of the inferred pattern of sequence diversity, placement within the gene network, and/or levels of gene expression. Whether or not these toxins play an active role in intraspecific competition remains unknown but merits further investigation. Transcriptome annotation highlighted toxin gene abundance and identified new metalloproteases and CRISP candidate toxin genes based on toxin sequences from other venomous taxa. The functional GO groupings identified transcripts that may be more abundant during acrorhagi inflation and expansion, which occurs during an aggressive encounter. Additionally, semantic similarities of some GO groupings identified transcripts which may behave synergistically with other toxin peptides. Our results provide a baseline for future RNAseq analyses to investigate the role that various peptides may play in aggressive intraspecific encounters.

## Methods

### Library prep, sequencing, cleanup, assembly, annotation

Polyps of *A. elegantissima* were collected from the intertidal zone in Coos Bay, Oregon in 2012 and kept alive in the lab in flow-through aquaria filled with artificial sea water. The anemones were observed daily to watch for any indications of aggressive behavior [11]. Once a polyp exhibited aggressive behavior, the polyp initiating the aggressive behavior was removed and placed in a smaller acclimation tank. Following a 15 minute acclimation, five dilated acrorhagi were removed with tweezers from the aggressive polyp and combined into a single 1.5 mL microcentrifuge tube for total RNA extraction. Four polyps not engaging in aggressive encounters were moved to a separate acclimation tank. From each of these polyps, 5–10 non-dilated acrorhagi were removed and combined in a single tube to represent the acrorhagi of non-aggressive polyps. The acrorhagi were flash frozen and 600 μL of Buffer RLT (QIAGEN) were added to the sample, along with several small (1.5 - 2 mm) ceramic beads (BioExpress). The tissue was macerated in the tube using a Mini-Beadbeater-8 (BioSpec Products) for 30 seconds on the “Homogenize” setting. After 30 seconds, the tube was placed on ice and visually inspected to verify that the tissue was homogenized. Total RNA was extracted following the RNeasy Mini Kit (QIAGEN) protocol.

RNA samples were quantified using the Qubit RNA BR Assay kit (Life Technologies) on a Qubit 2.0 Fluorometer (Life Technologies) and RIN scores were calculated using the Agilent RNA 6000 Nano kit (Agilent Technologies) on the BioAnalyzer (Agilent Technologies). First strand synthesis, library construction, and subsequent paired-end 100 base sequencing was conducted at the Nucleic Acid Shared Resource – Illumina Core, The Ohio State University, Columbus, OH, USA. For each transcriptome, reads were filtered using the program Trimmomatic [77] to remove adapters, leading and trailing low quality bases (using a sliding window greater than 3 bases), reads shorter than 36 bases in length, and reads that fell below an average quality score of 15 using a four base sliding window. The cleaned data were checked using the program FastQC [78] to ensure that low quality reads and regions were removed.

Raw data were assembled *de novo* Trinity [79] using default parameters. Transcripts were analyzed to determine the expected read count and proportion of each transcript relative to the gene (IsoPct). Expression levels for aggressive and non-aggressive polyp transcripts were determined in RSEM [80]. To adjust for differences in library size and skewed expression of transcripts, we used the metric ‘transcripts per million’ (TPM) for each transcriptome [81]. TPM is preferred over other expression metrics as it is independent of mean expressed transcript length, thus allowing for cross comparison between multiple samples [74,80,82]. Our TPM values do not permit a statistical analysis of differential gene expression, but instead provide metrics that can be compared between aggressive and non-aggressive polyp transcriptomes, while controlling for transcript size and discrepancies between sample preparation [74,80].

### Identification of candidate toxin genes

Bioinformatic processing of the transcriptomes used a combination of custom PERL scripts and sequence annotation programs. To identify candidate toxin genes, taxonomically diverse sea anemone toxin sequences were downloaded from GenBank (Additional file 2: Table S2). Candidate toxin genes from both transcriptomes were identified via the tBLASTn search algorithm (E value cutoff of 10). Matches from the BLAST searches were visually inspected for the key cysteine amino acid residues that are characteristic of the different venom types [50] and then screened for premature stop codons. We conducted a BLASTx search against the NCBI-nr protein database and BLASTn searches against the NCBI-nr nucleotide database and EST database to confirm that these sequences would retrieve toxin sequences. Protein sequences of candidate toxin genes were visualized in BioEdit [83] and aligned to known sea anemone toxin genes using ClustalW [84]. Protein sequences were screened for the placement of key cysteine amino acid residues [50]. Sequences that did not introduce large gaps (greater than variation observed in previously described proteins) into the alignments were retained for further processing (Additional file 5). The signaling region for each transcript (if present) was determined using SignalP [85]. Sequences with sequence identity of 90% or greater were considered isoforms of the same gene; however, sequences within gene networks with greater than 50% bootstrap support were also explored as potential divergent alleles.

To broaden our search for additional toxin genes beyond those previously identified in sea anemones, we used BLAST to search the transcriptomes of acrorhagi from aggressive and non-aggressive polyps against the UniProt ToxProt dataset [86], with sequences identified in the sea anemone toxin query removed. The candidate toxin-like transcripts were further processed by removing any redundant protein sequences and screened based on the following criteria: E value <1e-04 and identity percentage >20%. In addition to characterizing the overall taxonomic diversity associated with candidate toxin genes, we also looked at relative abundance of each of these genes and the presence of a signaling region which is characteristic of many toxin coding genes [67,71].

### Alignment and gene network reconstruction

Phylogenetic reconstruction provides a robust tool for identifying toxin genes [87]. We reconstructed unrooted gene networks for toxins identified as PLA2s, cytolysins, VGPC, and VGSC. Nucleotide sequences of candidate toxin genes and previously described sea anemone toxins were translated into protein sequences and aligned using default parameters in MAFFT (L-INS-i) [88]. Nucleotide alignments were created in BioEdit [83] using the protein alignment as a guide. Due to significant length variation and absence of the signaling/propeptide region in other taxa, gene networks were reconstructed with and without this region for the protein and nucleotide alignments (Additional file 5). As the majority of sea anemone toxins were acquired via mass spectrometry and thus lack the signaling/propeptide region, mature protein sequences of previously characterized toxin genes were incorporated into the final gene network reconstructions (Additional file 2: Table S1). MEGA [89] was used to select appropriate evolutionary models and reconstruct gene networks for both protein and nucleotide sequences (Additional file 2: Table S1). Protein and nucleotide datasets were subjected to 1000 rounds of bootstrap resampling in a maximum likelihood framework to determine branch support values for all toxin gene families.

### Transcriptome annotation and gene ontology

To assess the completeness of the transcriptome of acrorhagi from aggressive and non-aggressive polyps we used CEGMA (Core Eukaryotic Genes Mapping Approach) to identify the presence of 248 highly conserved proteins found across eukaryotes [72]. Annotation and homology searches of assembled transcripts from both transcriptomes were conducted using a suite of sequence annotation tools in the Trinotate pipeline [90]. Contrary to the hierarchical GO assignment approach of the popular program Blast2GO [91], Trinotate assigns transcripts among varying hierarchical levels within gene ontology (GO) networks. Free from hierarchical groupings, Trinotate permits cross comparisons across potentially functionally important genes that are found at different hierarchical levels within the gene ontology. Assembled transcripts were translated into their longest open reading frame peptide in TransDecoder [92]. Homology searches were conducted against SwissProt [93] using BLASTx searches of raw transcripts and BLASTp for the translated protein sequences. Conserved protein domains were identified using the program HMMR [75] against the Pfam domain database [94]. SignalP [85] was used to predict the signaling region of each transcript and tmHMM [95] was used to identify transmembrane regions. Functional annotation was performed by comparing BLAST hits with the annotated GO Pathways databases [96]. REVIGO [97] was used to visualize the GO group frequency among the transcripts with 0.9 similarity and all other parameters default.

### Availability of supporting data

Candidate toxin sequences used in our analyses are included as Additional file 1 and unmodified transcriptome assemblies on our data website (http://u.osu.edu/anemonedata/data/). All of the raw sequencing reads used to construct each transcriptome is available under BioProject accession PRJNA266623. The raw Illumina sequence reads have been deposited on NCBI’s SRA archive (Aggressive: SRR1645256, Non-Aggressive Polyp: SRR1646677) and the assembled transcriptomes on NCBI’s TSA database (Aggressive: GBXJ00000000, Non-Aggressive Polyp: GBYC00000000) that have been subjected to NCBI’s contamination screen.

## Additional files

Additional file 1:
**Nucleotide sequences of candidate toxin sequences identified in this study, includes both signaling and mature toxin when applicable.**
Additional file 2: Table S1. Parameters for toxin gene network reconstruction for various toxin candidates. **Table S2.** Genbank accession numbers for known anemone toxin sequences used in BLAST searches and subsequenet analyses. **Figure S1.** Protein sequence alignment of candidate type I VGPC toxins alongside previously characterized toxin proteins. **Figure S2.** Sequence alignments of Acrorhagin I and Acrorhagin II along with candidate toxin sequences identified from each transcriptome. Additional file 3:
**ToxProt BLAST hits and subsequent analysis for the aggressive polyp acrorhagi and the non-aggressive poyp acrorhagi transcriptomes.**
Additional file 4:
**Trinotate output for the aggressive polyp acrorhagi and the non-aggressive poyp acrorhagi transcriptomes.**
Additional file 5:
**Protein sequence alignments for all the toxin genes used in the toxin gene network reconstructions.**

## Abbreviations

RNA-seq: RNA sequencing
CRISP: Cysteine rich secretory protein
PLA2s: Phospholipase A2s
VGPC: Voltage gated potassium channel
VGSC: Voltage gated sodium channel
APETx2: Acid sensing channel toxin
BLAST: Basic local alignment search tool
GO: Gene ontology
CEGMA: Core eukaryotic genes mapping approach
ORF: Open reading frame
