# Supplementary material for: A RNA-seq approach to identify putative toxins from acrorhagi in aggressive and non-aggressive Anthopleura elegantissima polyps
Source: BMC Genomics. 2015 Mar 21;16(1):221. doi: 10.1186/s12864-015-1417-4 (PMC4397815; doi:10.1186/s12864-015-1417-4)
Supplement: Additional file 2: Table S1. — Parameters for toxin gene network reconstruction for various toxin candidates. Table S2. Genbank accession numbers for known anemone toxin sequences used in BLAST searches and subsequenet analyses. Figure S1. Protein sequence alignment of candidate type I VGPC toxins alongside previously characterized toxin proteins. Figure S2. Sequence alignments of Acrorhagin I and Acrorhagin II along with candidate toxin sequences identified from each transcriptome. [file 12864_2015_1417_MOESM2_ESM.pdf]

<u>Supplemental Table 1</u> Parameters for phylogenetic tree reconstruction. N = total number of sequences, L = Length of sequence alignment, l = Length of mature sequence alignment, Model = best DNA/Protein model of evolution determined by MEGA, AICc = Akaike Information Criterion, corrected value, # = number of sites used in analysis, log(LN) = log likelihood values of maximum likelihood trees, G/I = Gamma/I value for each tree. Dashes indicate incomplete data as no gene network was reconstructed.

Toxin	***N***	***L***	***l***	Model	AICc	#	log(LN)	G/I
Nucleotide								
**PLA2**	20	696	417	Kimura 2 (G+I)	93891.62	270	-4781.1428	1.5149/18%
**Cyto-II**	14	709	540	Kimura 2 (G)	8155.884	485	-3958.8144	0.8889/-
**VKSC**	24	327	201	Kimura 2 (G+I)	2743.201	139	-1502.9669	2.3786/15%
Protein								
**PLA2†**	83	189	168	WAG (G+I)	6611.927	32	-3205.730	1.5760/12%
**Cyto-II**	13	223	173	LG (G)	4143.187	161	-2038.9973	0.9244/-
**Cyto-IV**	4	467	-	-	-	-	-	-
**VGSC**	24	109	66	Dayhoff (+G)	1600.385	46	-879.1404	2.8514/-
**VGPC-II/KPI**	36	107	72	WAG (+G)	3804.086	50	-1823.0990	1.7589/-
**VGPC-III**	41	110	70	WAG (+G)	3096.826	31	-1258.6265	1.6220/-
**VGPC-IV**	4	41	-	-	-	-	-	-
**VGPC-V**	6	88	-	-	-	-	-	-
**VGPC-I**	37	100	53	WAG (+G)	1794.406	26	-817.9125	2.7421/-
**Acrorhagins-I**	4	93	-	-	-	-	-	-
**Acrorhagins-II**	5	64	-	-	-	-	-	-

Supplemental Table 2 Taxa included in this study, with GenBank accession numbers. Taxa are organized alphabetically for each toxin gene type. Bold GenBank accession numbers were used as query sequences.

**Phospholipase A2s**	**Genbank ID**
Adamsia carciniopados	**18033525**
Aedes aegypti	157137214
Aipysurus eydouxii	49472980, AAT66310
Alligator sinensis	XP_006019144
Bitis caudalis	P00622
Bos taurus	291575120
Bothrops asper	P24605.3
Branchiostoma floridae	260800263
Bungarus multicinctus	CAD24463
Caenorhabditis elegans	3874090
Callorhinchus milii	XP_007906926
Canis lupus familiaris	50979154
Ciona intestinalis	198415032
Condylactis gigantea	**283140837**
Cryptophis nigrescens	ABK63571
Culex quinquefasciatus	170048777
Demansia vestigiata	118151758, ABK63569
Dicentrarchus labrax	4454121
Equus caballus	149720537
Gallus gallus	363740066
Heterocephalus glaber	XP_004843730
Homo sapiens	4505847
Hoplocephalus stephensii	124020979
Mus musculus	12842453
Nasonia vitripennis	156540704
Nematostella vectensis	156339845, 156391018, 156391017, 156391019, 156391020, 156365766, 156365765, 156383321, 156378642, 156383865, 156383365
Notechis scutatus	71066758, 71066760
Oxyuranus scutellatus	71066728, AAZ22637, AAB33760, AAZ22635, Q45Z47.2
Oxyuranus scutellatus scutellatus	AAY47071, AAY47070
Pagrus major	2662295, 13928533, BAB20041
Patiria pectinifera	5902462
Pseudechis australis	71066788
Pseudechis porphyriacus	AAZ22670
Rattus norvegicus	13928792
Strongylocentrotus purpuratus	390366769
Sus scrofa	51592123
Sycon raphanus	AM765083
Taeniopygia guttata	XP_004175830
Trichoplax adhaerens	196012902, 196012904, 196012906
Urticina crassicornis	**152003427**
Xiphophorus maculatus	XP_005811202
**Cytolysins**	
Actinia equina	**FM958450, U41661, U51900, AF057028**
Anthopleura asiatica	**AB479475**
Heteractis crispa	**AY855350**
Oulactis orientalis	**AY861662**
Sagartia rosea	**AY247033**
Stichodactyla gigantea	**JQ353486**
Stichodactyla helianthus	**AJ005038, AJ009931**
Urticina crassicornis	**GQ848199**
**VGPC type I Toxins**	
Actinia equina	**P81897**
Anemonia erythraea	**Q0EAE5**
Anemonia sulcata	**Q9TWG1**
Cryptodendrum adhaesivum	**BAJ23161**
Heteractis magnifica	**AAD09480**

Supplemental Table 2
(Continued)

**VGPC type I Toxins *(Continued)***	**Genbank ID**
Heterodactyla hemprichii	**BAJ23162**
Metridium senile	**P11495**
Stichodactyla mertensii	**BAJ23160**
Stichodactyla haddoni	**BAJ23159**
Stichodactyla gigantea	**BAJ23158**
Thalassianthus aster	**BAJ23163**
**VGPC type II Toxins**	
Anemonia sulcata	**Q9TWF8, Q9TWG0, Q9TWF9**
Stichodactyla helianthus	**B1B5I8**
**Kunitz-Type Protease Inhibitors**	
Actinia equina	**P0DMJ2**
Anemonnia sulcata	**P10280**
Anthopleura elegantissima	**P86862**
Anthopleura fuscoviridis	**P0DMJ3, P0DMJ4**
Anthopleura xanthogrammica	**P81548, P81547**
Heteractis crispa	**P16344**
Stichodactyla helianthus	**13M7Q_B, P81129**
**VGPC type III Toxins**	
Anemonia sulcata	**P11494, P59084**
Anemonia viridis	FK744472, FK722457, FK736010, FK723172, FK745823, FK754940, FK721972, FK740326, FK725608, FK752236, FK720902, FK736435
Antheopsis maculata	**P69930**
Anthopleura elegantissima	**P61542, P61541,** B3EWF9
Bunodosoma cangicum	P86461, P84919
Bunodosoma granuliferum	CCC86602, CCC86603, CCC86604, CCC86606
**VGPC type IV Toxins**	
Stichodactyla haddoni	**P0C7W7**
**VGPC type V Toxins**	
Bunodosoma caissarum	**C0HJC4**
**VGSC type I,II,III Toxins**	
Actinia equina	**ABW97361, ABW97358, ABW97362, Q9NJQ2**
Aiptasia diaphana	**ACQ83467**
Anemonia erythraea	**P69943**
Anemonia viridis	**FK728784, FK754096, P01533, P0C7Q0, ABW97326, P01529**
Antheopsis maculata	**P69928**
Anthopleura elegantissima	**P0C1F0, P0C1F1, P01532, P0C1F3**
Anthopleura fuscoviridis	**P10453**
Anthopleura sp.	**P0C5F4, P0C5F5, P0C5F6, P0C5F7**
Anthopleura xanthogrammica	**P0C5F8, P0C5G1, P0C5G2, P0C5G3, P01530, AAA27737, P0C5F9, P0C5G0**
Bunodosoma cangicum	**P86460, P86459, P0C7P9, P82803, Q7M425**
Bunodosoma granuliferum	**P0C1F4, P0C1F5**
Calliactis parasitica	**P49127, P14531**
Condylactis gigantea	**P0C280**
Condylactis passiflora	**P0CH42**
Cryptodendrum adhaesivum	**D2KX90**
Halcurias carlgreni	**P0C5G6**
Heteractis crispa	**P30785, P30832, P30784, P30783, P30831, P0C5G5**
Heterodactyla hemprichii	**D2KX91**
Nematostella vectensis	**ABW97340, ABW97343, ABW97332, ACB 71119, ABW97348, ACB 71121**
Radianthus paumotensis	**P08380, P01534**
Stichodactyla gigantea	**Q76CA0**
Stichodactyla gigantea	**Q76CA3**
Stichodactyla haddoni	**B1B5I9**
Thalassianthus aster	**D2KX92**
**Acrorhagins**	
Actinia equina	**BAE46981, BAE46982, BAE46983, BAE46984**

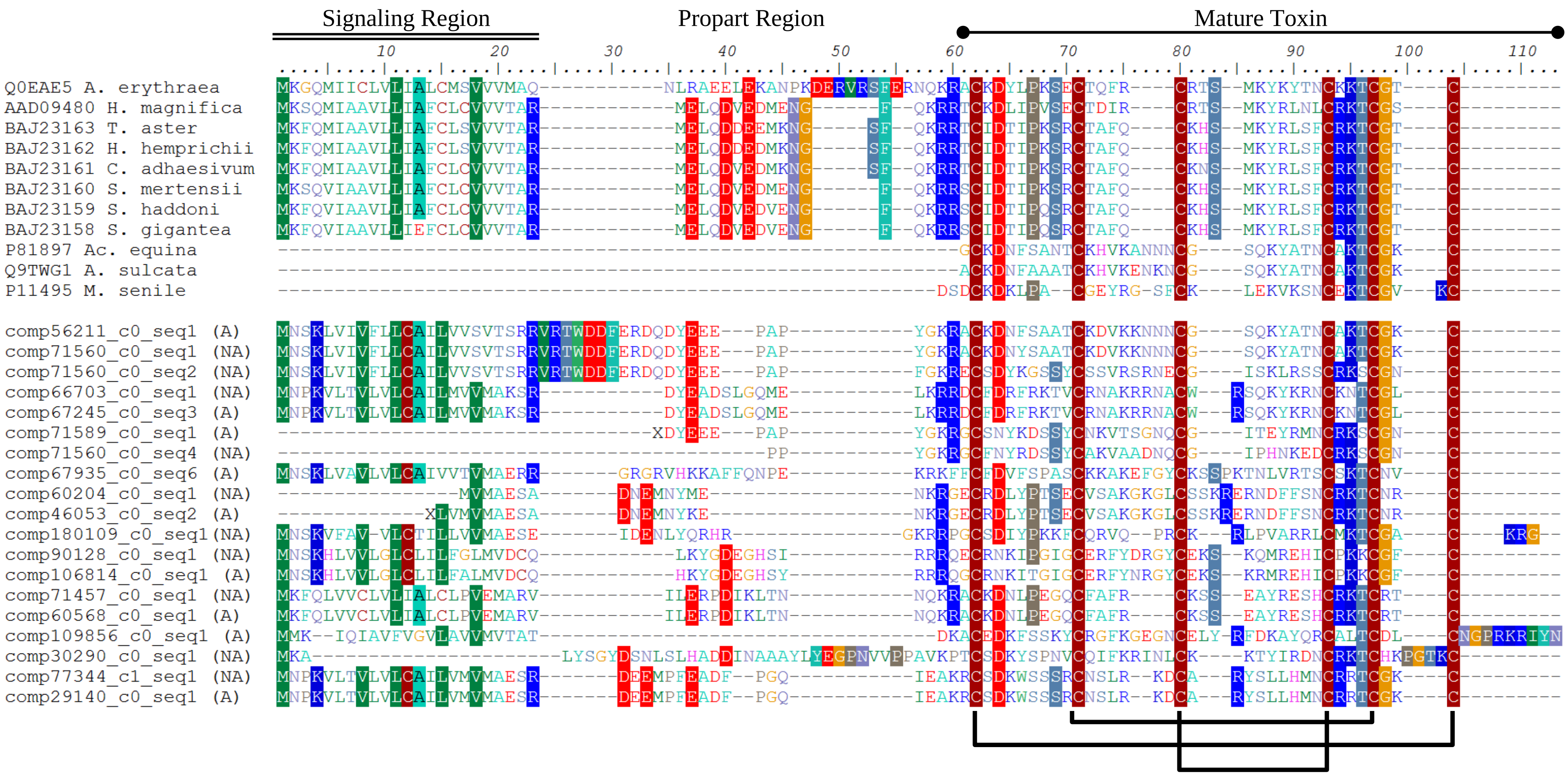

Supplemental Figure 1 Type I VGPC toxin sequence alignment of previously described toxins and candidate toxin genes. The identification of the signaling, propart, and mature regions is based on previously published sequences. The placement of the cysteine bonds forming the disulfide backbone is indicated in brackets. The transcriptome source is indicated (acrorhagi from A: aggressive or NA: non-aggressive polyps). For full species name refer to S. Table 2. Residues with >50% similarity have the color background shaded in.

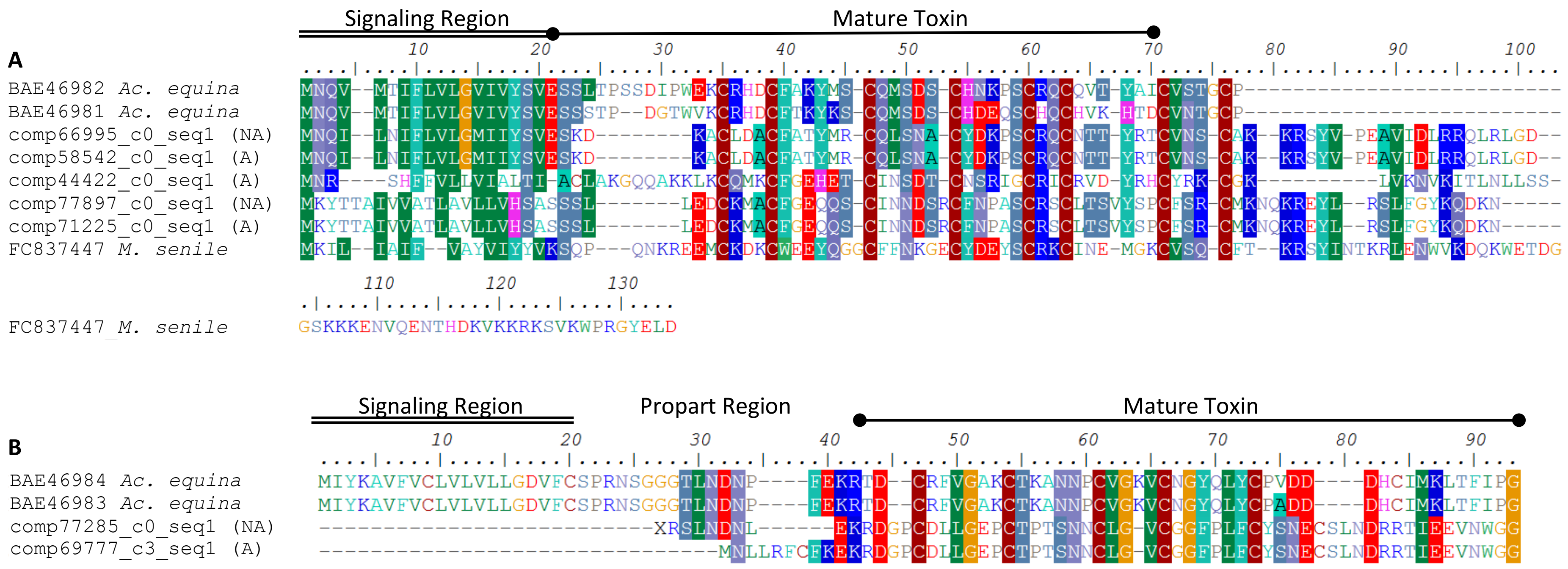

Supplemental Figure 2 Sequence alignments of Acrorhagin I (A) and Acrorhagin II (B). The signaling, propart, and mature toxin regions are based on previously published sequences. The transcriptome source is indicated (acrorhagi from A: aggressive or NA: non-aggressive polyps). Residues with >50% similarity are colored in.
